# Supplementary material for: Distinct population dimensions separate sensory adaptation and expectation-related signals in visual cortex
Source: bioRxiv. 2026 Oct 2:2026.09.30.755465. Preprint. [Version 1] doi: 10.64898/2026.09.30.755465 (PMC13648692; doi:10.64898/2026.09.30.755465)
Supplement: Supplementary file 1 [file media-1.pdf]

Supplementary Information

Distinct population transformations underlie visual mismatch responses in V1

Supplementary Figures and Legends

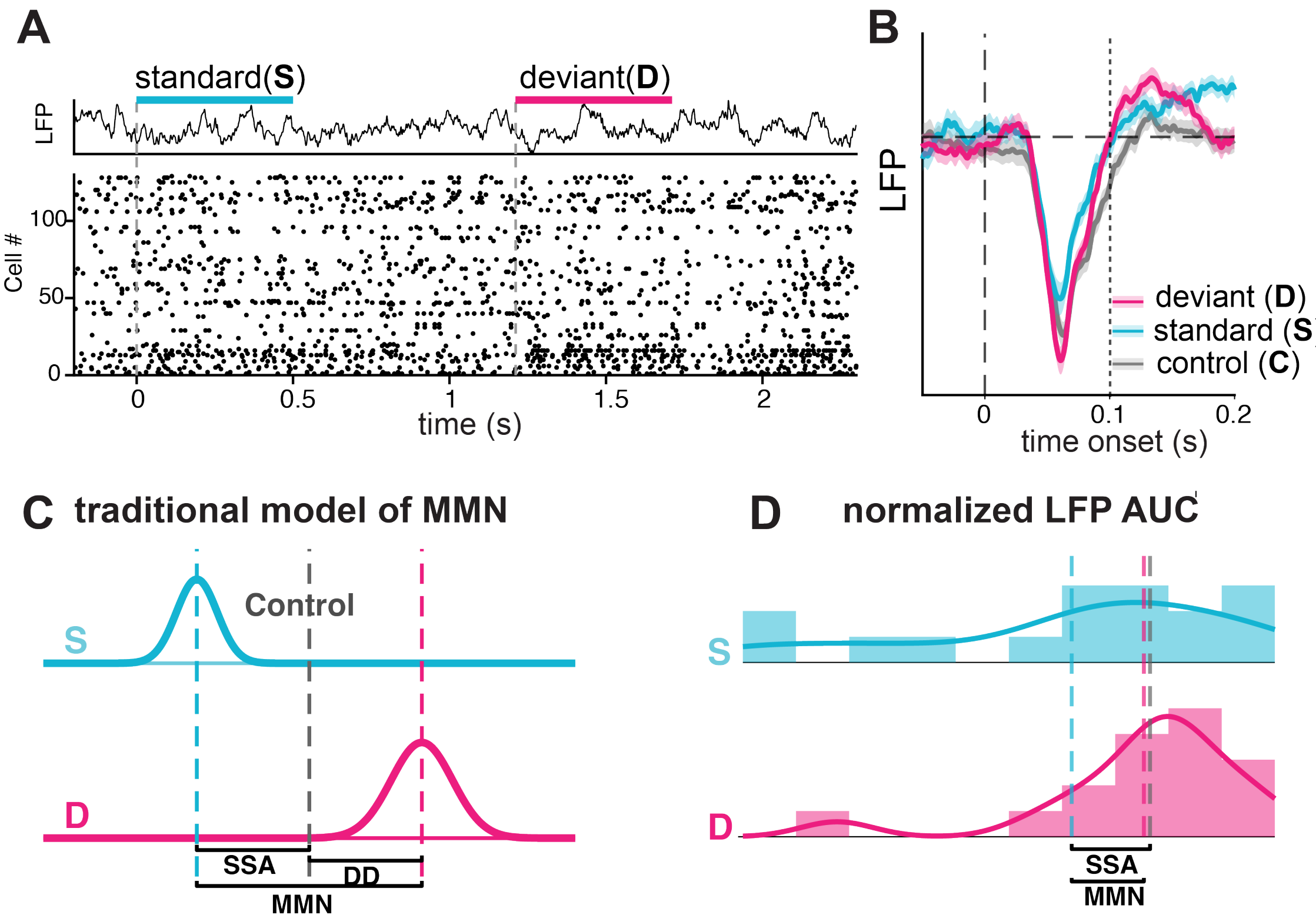

Figure S1. LFP responses provide an aggregate reference for visual mismatch. A. Conventional scalar model in which $C-S$ is termed stimulus-specific adaptation (SSA), $D-C$ is termed deviance detection (DD), and $D-S$ is the mismatch (MM) contrast. **B.** Normalized LFP response magnitude for standard, control, and deviant stimuli. **C.** Example standard and deviant LFP time courses. **D.** Summary of LFP responses. LFP measurements capture aggregate differences among presentation conditions but do not resolve the population geometry examined in the main figures.

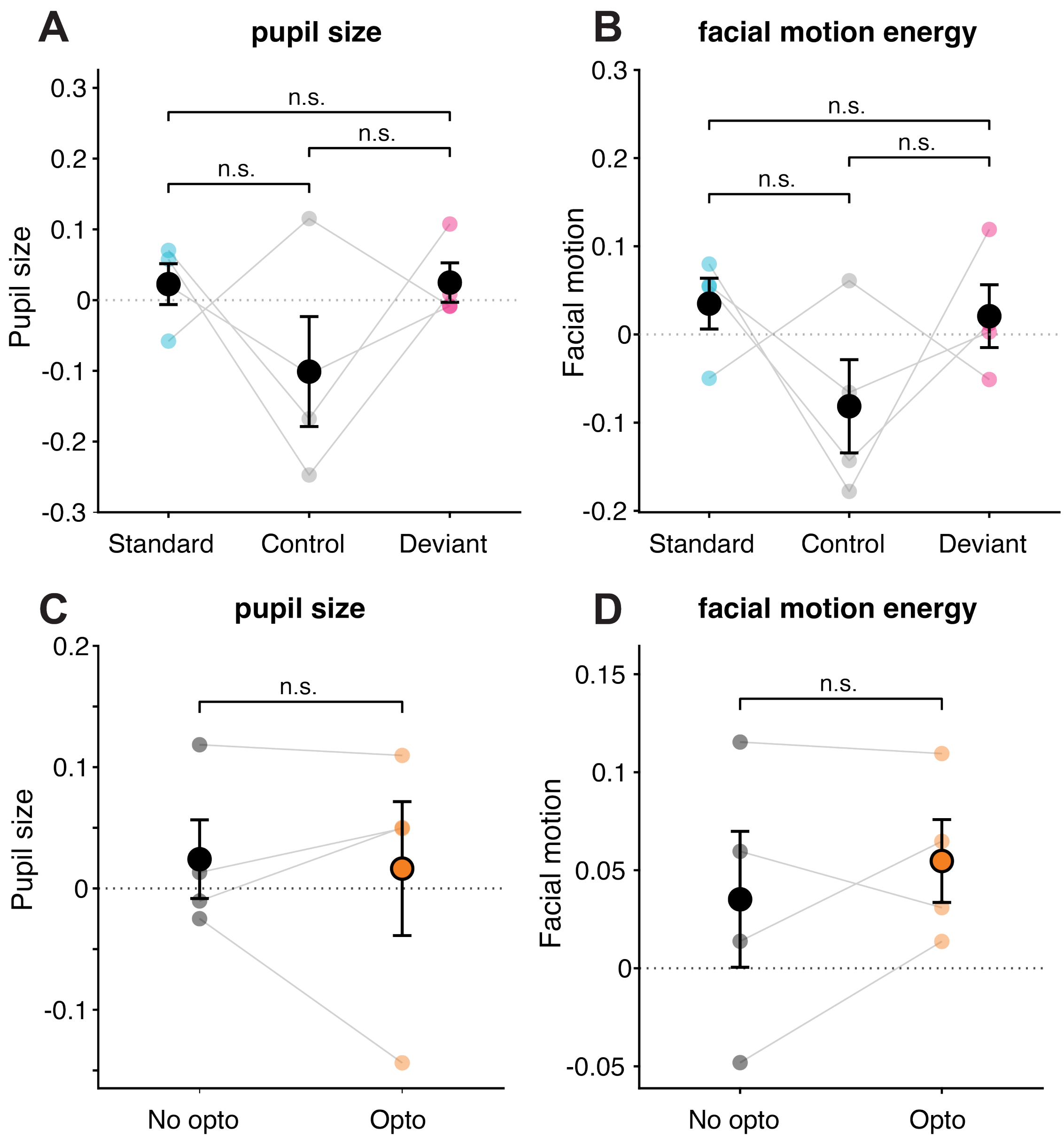

Figure S2. Measured behavioral state across visual-stimulus and optogenetic conditions. A,B. Trial-averaged pupil size (**A**) and facial motion energy (**B**) during standard, control, and deviant trials without optogenetic illumination. No significant differences were detected among stimulus conditions ($n = 4$ mice; two-sided paired tests: pupil size, S versus C, $p = 0.326$; C versus D, $p = 0.267$; S versus D, $p = 0.932$; facial motion energy, S versus C, $p = 0.245$; C versus D, $p = 0.318$; S versus D, $p = 0.547$). **C,D.** Pupil size (**C**) and facial motion energy (**D**) during ACC-terminal-inhibition and no-inhibition trials in the oddball paradigm. Neither measure differed significantly between optogenetic conditions ($n = 4$ mice; two-sided paired tests: pupil size, $p = 0.195$; facial motion energy, $p = 0.438$). Points indicate individual mice; summary values indicate mean ± SEM.
